# Perceived Work Environment, Professional Identity, and Dispositional Hope Among Nurse Interns: A Cross-Sectional Study

**DOI:** 10.3390/healthcare14172683

**Published:** 2026-08-24

**Authors:** Marwa Abd Elrahman Gaber Khalifa, Rasha Kadri Ibrahim, Abdelaziz Hendy, Nada Alqarawi, Fawzia Mohamed Mohamed Badran, Eman Hassan Mohamed Ali, Shaimaa Mahamed Araby Ebraheem

**Affiliations:** 1 Nursing Administration Department, Faculty of Nursing, Ain Shams University, Cairo 11517, Egypt; dr.marwa.khalifa@nursing.asu.edu.eg (M.A.E.G.K.); dr.eman.ali@nursing.asu.edu.eg (E.H.M.A.); 2 Nursing Department, Fatima College of Health Sciences, Al Dhafra Region, Madinat Zayed 23647, United Arab Emirates; 3 Department of Maternal and Child Health Nursing, College of Nursing, Qassim University, Buraydah 52571, Saudi Arabia; a.hendy@qu.edu.sa; 4 Department of Community, Psychiatric, and Mental Health Nursing, College of Nursing, Qassim University, Buraydah 52571, Saudi Arabia; 5 Nursing Services Department, Qassim University Medical City, Buraydah 52571, Saudi Arabia; 6 Nursing Administration Department, Faculty of Nursing, Benha University, Benha 13511, Egypt

**Keywords:** nurse interns, professional identity, perceived work environment, hope, transition to practice, clinical practice

## Abstract

Background: The transition from nursing education to professional practice requires nurse interns to consolidate clinical competence, develop professional identity, and establish future expectations within their work environments. Aim: This study examined the associations among perceived work environment, professional identity, and dispositional hope among nurse interns and assessed whether perceived work environment and professional identity remained independently associated with dispositional hope after adjustment for demographic and internship-related characteristics. Methods: An analytical cross-sectional correlational study was conducted among 1450 nurse interns at Benha University Hospitals, Egypt, using a total population sampling approach. Data were collected using the Perceived Work Environment Questionnaire, Professional Identity Scale for Nursing Students, and Adult Hope Scale. Descriptive statistics, Pearson’s correlation, and multiple linear regression were performed. Results: The mean scores were 138.42 ± 18.92 for perceived work environment, 57.84 ± 8.96 for professional identity, and 47.20 ± 5.68 for dispositional hope. Perceived work environment showed significant positive correlations with professional identity (r = 0.58, *p* < 0.001) and dispositional hope (r = 0.52, *p* < 0.001), while professional identity was also positively correlated with dispositional hope (r = 0.49, *p* < 0.001). In the multiple linear regression analysis, perceived work environment (β = 0.359, *p* < 0.001) and professional identity (β = 0.282, *p* < 0.001) were both positively associated with dispositional hope after adjustment for the included demographic and internship-related covariates. The overall regression model was statistically significant—F (10, 1439) = 70.054, *p* < 0.001—and explained 32.7% of the variance in dispositional hope (R^2^ = 0.327; adjusted R^2^ = 0.323). Conclusions: Perceived work environment and professional identity were independently associated with dispositional hope among nurse interns. These findings underscore the relevance of structured supervision, constructive feedback, mentoring, and professional identity development within nursing education and internship programs.

## 1. Introduction

Nursing is a demanding profession characterized by continuous exposure to stressful situations and a persistent global workforce shortage [1]. These challenges are particularly pronounced during the internship year, when nurse interns transition from education to professional practice, consolidate clinical competence, apply theoretical knowledge, and develop their professional identity [2,3,4,5].

Within this transitional period, the perceived work environment is associated with nurse interns’ learning and professional development. Effective supervision, positive psychosocial climates, meaningful learning opportunities, and collaborative relationships enhance empowerment, engagement, and preparedness for professional practice [4,6,7,8,9]. Similarly, active participation, inclusion within healthcare teams, and appropriate learning opportunities facilitate interns’ integration into clinical practice [10].

Beyond supporting clinical learning, the quality of the work environment is associated with clinical performance, job satisfaction, professional socialization, and commitment to nursing [7,9,11]. In contrast, inconsistencies between educational preparation and clinical practice reduce engagement and confidence [12,13]. Positive clinical experiences are also associated with professional identity development during the internship year [14,15,16].

Professional identity reflects the internalization of professional values, roles, and responsibilities during the transition to practice [15,17,18]. A strong professional identity is associated with greater self-confidence, professional satisfaction, and performance, whereas a weak professional identity is associated with stress and reduced professional engagement [7,18,19]. Accordingly, leadership, mentorship, and positive role modeling are central to this developmental process. Through authentic clinical experiences, clinical educators and preceptors facilitate interns’ integration into practice and internalization of professional values [20,21,22,23].

Alongside professional identity, dispositional hope supports motivation and goal-directed behavior during the transition to practice. Snyder’s Hope Theory defines hope through agency, representing goal-directed motivation, and pathways, representing planning to achieve goals [24]. Higher dispositional hope is associated with resilience, emotional well-being, career planning, and professional identity, whereas lower hope is associated with psychological distress and reduced coping capacity [4,25,26,27].

Collectively, previous research has established relationships among the perceived work environment, professional identity, and hope among nurse interns. Building on this evidence, the present study examines perceived work environment, professional identity, and hope simultaneously and determines whether perceived work environment and professional identity are independently associated with hope after adjustment for demographic and internship-related characteristics. Given its cross-sectional design, the study examines associations without inferring causal relationships.

### 1.1. Theoretical Framework

Snyder’s Hope Theory and Social Cognitive Theory provide complementary explanations for the relationships among perceived work environment, professional identity, and hope. Hope Theory conceptualizes hope in terms of agency and pathways. Agency represents goal-directed motivation, whereas pathways represent the capacity to identify routes for achieving goals. During the transition to practice, a supportive work environment provides resources, feedback, and learning opportunities that are associated with nurse interns’ professional goals and future expectations.

Extending this perspective, Social Cognitive Theory explains the reciprocal interaction between personal factors and the work environment [28]. Guidance, professional interactions, role modeling, and participation in clinical practice support the internalization of nursing values, roles, and responsibilities. These processes link the perceived work environment to professional identity. In turn, professional belonging and commitment strengthen motivation and future-oriented thinking, linking professional identity to hope.

Together, the theories connect environmental experiences, professional development, and goal-directed thinking. Social Cognitive Theory explains how clinical experiences relate to professional identity, while Hope Theory explains the motivational and cognitive processes underlying hope. The framework also recognizes reciprocal relationships because professional identity and hope can influence how nurse interns perceive their work environment. Although professional identity can theoretically mediate the relationship between perceived work environment and hope, simultaneous cross-sectional measurement does not establish temporal precedence. Therefore, the study treats perceived work environment and professional identity as concurrent correlates of hope without asserting causal relationships.

### 1.2. Aim of the Study

This study aimed to examine the associations among perceived work environment, professional identity, and dispositional hope among nurse interns at Benha University Hospitals. Additionally, the study aimed to examine the relative associations between the perceived work environment, professional identity, and dispositional hope and to determine whether these associations remained significant after adjustment for demographic and internship-related characteristics.

### 1.3. Research Hypothesis

**H1.**  *Perceived work environment is positively associated with professional identity among nurse interns.*

**H2.**  *Perceived work environment is positively associated with dispositional hope among nurse interns.*

**H3.**  *Professional identity is positively associated with dispositional hope among nurse interns.*

**H4.**  *Perceived work environment and professional identity are independently associated with dispositional hope among nurse interns after adjustment for selected demographic and internship-related characteristics.*

## 2. Methods

### 2.1. Study Setting and Design

This analytical, cross-sectional, correlational study was conducted at Benha University Hospitals in Egypt. These hospitals are university-affiliated facilities providing secondary and tertiary care services to the surrounding community. They have 600 beds in total. Benha University Hospitals has three buildings. The Surgical Building includes 10 units and has 160 beds. The Medical Building has 24 units and has 430 beds. Finally, the Ophthalmology Building has 10 beds distributed in 2 units.

### 2.2. Participants and Sampling Strategy

The target population for this study included nurse interns at Benha University Hospitals for the 2024/2025 internship year. The study included nurse interns who were participating in the clinical internship during data collection and had adequate exposure to the perceived work environment. Adequate exposure was defined as active engagement in clinical rotations at the hospital, with regular attendance at clinical work and participation in supervisory and work-related activities. This activity would enable the interns to articulate their perceptions about the work environment. Also excluded were nurse interns who postponed their internship, repeated their internship year, were on leave during the data collection, or were absent during the questionnaire administration.

Participation was voluntary, and informed consent was documented for each participant. Data collection was preceded by informing participants that their data would be handled with strict confidentiality and that neither participation nor withdrawal would affect their academic progress or internship status.

A total of 1620 nurse interns met the eligibility criteria during the study period. Of these, 1530 questionnaires were returned, corresponding to a questionnaire return rate of 94.4%. Eighty returned questionnaires contained incomplete responses and were excluded from the analysis. The remaining 1450 fully completed questionnaires were included in the final analysis, representing an analyzable response rate of 89.5% of the eligible population.

### 2.3. Sample Size

Sample size was estimated a priori using G*Power software version 3.1.9.7 (Heinrich Heine University Düsseldorf, Düsseldorf, Germany), based on the guidelines proposed by Cohen [29] and Faul et al. [30]. The calculation was performed for multiple linear regression analysis (fixed model, R^2^ deviation from zero), assuming a medium effect size (f^2^ = 0.15), a significance level of α = 0.05, statistical power of 0.90, and ten variables. The estimated minimum required sample size was 147 participants. The final sample of 1450 nurse interns substantially exceeded the required sample size, providing adequate statistical power for the regression analyses.

The division of nurse interns into different clinical units and training areas added variety and increased sample representation across clinical training. Even though voluntary participation introduced some selection bias, numerous attempts were made to recruit interns from various work units to mitigate it. These participants were retained in the sample as no changes were made to the instruments or data collection methods after the pilot study.

### 2.4. Instruments

#### 2.4.1. Personal and Job Characteristics

A structured questionnaire was developed by the researchers to collect participants’ demographic and internship-related characteristics. The collected data included age, gender, marital status, residence, and current clinical training area.

#### 2.4.2. Perceived Work Environment Questionnaire

The perceived work environment among nurse interns was assessed using the 43-item Perceived Work Environment Questionnaire developed by Mohan and Lone [31], based on the instrument originally proposed by Smerek and Peterson [32]. The questionnaire was originally conceptualized within an employee work-environment context and was selected because of its established psychometric properties and its coverage of multiple organizational, interpersonal, and work-related aspects of the workplace.

The questionnaire comprises 43 items distributed across 12 dimensions: satisfaction with the job, satisfaction with recognition, satisfaction with work, satisfaction with opportunities for promotion, satisfaction with opportunities for professional advancement, satisfaction with responsibility, satisfaction with the organization, satisfaction with clarity of the mission, satisfaction with relationships with co-workers, satisfaction with supervision, satisfaction with salary, and satisfaction with core values. All items are rated on a 5-point Likert-type scale ranging from 1 = strongly disagree to 5 = strongly agree. According to the established scoring procedure of the instrument, responses across all 43 items are summed to obtain an overall perceived work environment score, with higher scores indicating more favorable perceptions of the work environment.

Although the questionnaire was originally developed within an employee context, several of its domains are conceptually relevant to nurse interns because interns undertake sustained clinical practice within hospital units and are directly exposed to supervision, relationships with co-workers, recognition, assigned responsibilities, organizational mission and values, and opportunities for professional learning and development. Nevertheless, some dimensions, particularly salary, opportunities for promotion, satisfaction with the organization, and opportunities for professional advancement, were originally conceptualized in relation to formally employed personnel and may have a different or more limited meaning for nurse interns. Accordingly, findings relating to these employment-oriented dimensions should be interpreted cautiously as reflecting aspects of the broader organizational environment experienced during internship rather than formal employment conditions.

Because English is the official language of instruction for nursing programs at Benha University, the questionnaire was administered in English. During the pilot study, the clarity and comprehensibility of the questionnaire items were assessed among nurse interns, and no modifications to the instrument were considered necessary. However, this assessment of clarity and comprehensibility was not considered a formal evaluation of content validity for the nurse intern population.

The factorial structure of the questionnaire in the present sample was examined using exploratory factor analysis (EFA). The suitability of the data for factor analysis was assessed using the Kaiser–Meyer–Olkin (KMO) measure of sampling adequacy and Bartlett’s test of sphericity. Principal axis factoring with oblique Promax rotation was used because correlations among the dimensions were expected. Factors were retained based on eigenvalues greater than 1, inspection of the scree plot, and theoretical interpretability. A 12-factor solution corresponding to the proposed dimensions of the questionnaire was retained and accounted for approximately 68.1% of the total variance. Factor loadings ranged from 0.56 to 0.84, and no substantial cross-loadings were observed. The KMO value was 0.92, and Bartlett’s test of sphericity was statistically significant (χ^2^ = 18,456, df = 903, *p* < 0.001). These findings supported the multidimensional factorial structure of the questionnaire in the present sample.

Although the EFA supported a 12-factor structure, the use of the total perceived work environment score in the primary analyses was not intended to imply that the instrument is unidimensional. Rather, the total score was used as an overall composite summary of the broader perceived work environment, consistent with the established scoring procedure of the questionnaire. This approach was also aligned with the primary aim and hypotheses of the study, which focused on the association of overall perceived work environment with professional identity and dispositional hope rather than on the independent associations of each individual work-environment dimension.

The instrument demonstrated excellent internal consistency, with Cronbach’s alpha coefficients ranging from 0.86 to 0.95 for the subscales and 0.91 for the total score. The high internal consistency of the total score supports the reliability of the composite measure in the present sample but should not be interpreted as evidence that the questionnaire is unidimensional. Similarly, the observed factorial structure and internal consistency do not establish content validity of the questionnaire specifically for nurse interns. Therefore, the 12 dimensions should be regarded as distinct but related components contributing to a broader composite representation of the perceived work environment, and interpretation of the overall score should recognize that aggregation may obscure potentially important domain-specific differences.

#### 2.4.3. Professional Identity Scale for Nursing Students (PISNS)

The Professional Identity Scale for Nursing Students (PISNS) was developed by Hao et al. [33]. The PISNS was used to understand nurse interns’ perceptions of the development of their professional identities during their transition to nursing practice. The PISNS has 17 items and 5 factors. These factors include professional self-image (6 items), retention and turnover risk (4 items), social comparison and self-reflection (3 items), independence of career choice (2 items), and social modeling (2 items). The items of the PISNS are scored using a 5-point Likert scale. The scores for the PISNS are summed to obtain a total score, which ranges from 17 to 85. The higher the score, the stronger the respondents’ professional identity.

The PISNS was administered in English because the participants’ nursing education was conducted in English. In the current study, the PISNS demonstrated excellent internal consistency, with a Cronbach’s alpha of 0.95 for the total scale. Reliability analysis was also conducted for each subscale, demonstrating acceptable to excellent internal consistency across the five dimensions: professional self-image (α = 0.91), retention and turnover risk (α = 0.88), social comparison and self-reflection (α = 0.86), independence of career choice (α = 0.82), and social modeling (α = 0.84). These findings support the reliability of both the overall PISNS score and its individual dimensions in the current sample of nurse interns.

#### 2.4.4. Adult Hope Scale (AHS)

Hope was assessed using the Adult Hope Scale (AHS), developed by Snyder et al. [24], based on Snyder’s cognitive theory of hope. The scale measures dispositional hope through two dimensions: agency, representing goal-directed motivation, and pathways, representing perceived ability to generate strategies for achieving goals. The AHS consists of 12 items, including four filler items that are not scored. The remaining eight items are equally distributed between the agency and pathways subscales. Responses are rated on an 8-point Likert scale ranging from 1 (definitely false) to 8 (definitely true). The total score ranges from 8 to 64, with higher scores indicating higher levels of dispositional hope. The instrument was administered in English because English is the language of nursing education at Benha University.

In the present study, we assessed the construct validity of the Adult Hope Scale (AHS) and the extension of its theoretical framework, comprising the agency and pathways dimensions, to the Egyptian nurse interns. To this end, the two-dimensional structure was subjected to a confirmatory factor analysis (CFA). The proposed structure was shown to provide an acceptable fit (χ^2^/df = 2.31, CFI = 0.96, TLI = 0.95, RMSEA = 0.05, and SRMR = 0.04) with all of the items having adequate factor loadings (given that the agency and pathways dimensions’ estimated factor loadings were in the interval of 0.62 to 0.84 and 0.59 to 0.82, respectively). The two dimensions were shown to be significantly correlated (r = 0.68, *p* < 0.001) and to be related yet separable constructs of dispositional hope. The analysis of the subscale scores revealed that the agency and pathways dimensions of the AHS had adequate internal consistency with Cronbach’s alphas of 0.86 and 0.84, respectively, with McDonald’s omegas of 0.87 and 0.85, respectively. In the current study, the scale also exhibited adequate internal consistency, with the AHS total score having a Cronbach’s alpha of 0.93. In light of these findings, the AHS is a valid and reliable measure of dispositional hope of Egyptian nurse interns.

### 2.5. Data Collection Procedure

Data collection commenced following administrative approval from the Benha University Faculty of Nursing and the corresponding hospital units and continued for two months, from July 2025 to August 2025.

A pilot study with 145 nurse interns, approximately 10% of the anticipated sample size, was used to test the clarity, practicality, and relevance of the proposed study. The study also aimed to estimate the questionnaire completion time, which was found to be between 25 and 35 min. Clarity of the study items was affirmed by the study participants, and no further changes were required to the study tools or data collection procedures. Participants of the pilot study became study participants. The participants reported that the items were clear and understandable, and no modifications were required. Therefore, the pilot study participants were included in the final study sample of 1450 nurse interns.

In the subsequent study, researchers collaborated with the head nurses of the participating clinical units to identify the most appropriate times to administer the study questionnaires to ensure that the nurse interns’ clinical duties were not disrupted. Researchers described the study purpose and the administration procedures and emphasized that participation was voluntary prior to administering the questionnaires to nurse interns across the various clinical shifts. Researchers remained present to address any concerns regarding the questionnaire items while ensuring that participants were not influenced in any way. The interns were informed of their right to volunteer/withdraw at will and that their choice to do so would not affect their evaluation, internship placement, or employment opportunities. Their choice to volunteer was further protected by ensuring the confidentiality of their responses. This was accomplished by anonymizing the questionnaires and maintaining anonymity throughout the data collection and analysis phases.

### 2.6. Ethical Considerations

Prior to beginning data collection, ethical approval (Approval No. REC-NA-P81) was granted by the Scientific Research Ethics Committee of the Faculty of Nursing, Benha University, Egypt. The study was compliant with the Declaration of Helsinki. Nurse interns received detailed information about the study, including its purpose, procedures, potential benefits, and rights, prior to participation. Written informed consent was obtained from all nurse interns before completing the questionnaire. Participation was voluntary, and nurse interns were fully informed that declining to participate or withdrawing from the study at any time would not negatively affect their academic, administrative, or professional standing. The study maintained ethical research standards of anonymity and confidentiality. Coded questionnaires were utilized in place of personal identification, and no personal information was collected. The research team handled all data securely. The data collected were for the study’s purposes only.

### 2.7. Statistical Analysis

Data were coded, entered, and analyzed using IBM SPSS Statistics for Windows, version 26.0 (IBM Corp., Armonk, NY, USA). Descriptive statistics were used to characterize participants and study variables. Scores of the perceived work environment, professional identity, and dispositional hope were measured by the means and standard deviations, and the ranges of the scores were provided. Frequency and percent distribution were computed for categorical data.

The internal consistency of the study instruments was assessed using Cronbach’s alpha coefficients. Inferential statistics reliability was preceded by the assessment of continuous variables’ distribution. Assessment was done via graphic illustration methods that included the use of histograms and Q–Q plots, in addition to the conduct of normality tests (the Kolmogorov–Smirnov and the Shapiro–Wilk Tests). Given the study’s large sample size, normality tests were reported, but regression assumptions were assessed using residual diagnostics.

The relationships among the perceived work environment, professional identity, and dispositional hope were assessed using Pearson’s correlation coefficient (r). Multiple linear regression was conducted to assess the adjusted associations between professional identity and perceived work environment and dispositional hope, with relevant demographic and internship-related variables as control variables.

In the multiple linear regression analysis, dispositional hope was entered as the dependent variable. Perceived work environment and professional identity were included as continuous predictors using their original total scores. Age was represented using indicator variables, with participants aged 22–24 years as the reference category and separate indicators for those aged <22 years and >24 years. Gender was coded as male versus female, marital status as married versus single, and residence as rural versus urban. Clinical training area was represented using indicator variables, with maternal and newborn health nursing as the reference category and separate indicators for medical-surgical nursing, critical care, and dialysis/emergency nursing. These demographic and internship-related variables were included as covariates to examine the adjusted associations of perceived work environment and professional identity with dispositional hope. These covariates were selected based on their theoretical relevance and their potential associations with psychological and professional outcomes among nurse interns. Regression diagnostics were performed to assess the adequacy of the multiple linear regression model. Multicollinearity was evaluated using the variance inflation factor (VIF) and tolerance values.

To test the independence of the residuals, the Durbin–Watson statistic was 1.92, indicating that the errors were approximately independent. The assumption of linearity was tested using a scatter plot of standardized residuals versus model-estimated values, which showed an approximately linear relationship. The normality of the residuals was assessed using a histogram and a normal probability (P-P) plot, which showed an acceptable approximation to normality. Homoscedasticity was evaluated by inspecting the residual plots, which showed a fairly constant variance across the model-estimated values. The assessment of influence was conducted using Cook’s distance and leverage. The Cook’s distance values were below 0.05, indicating that no individual observation had a considerable influence on the regression estimates.

Since all study variables were measured using the same self-reporting questionnaires at the same point in time, common method bias was possible and taken into consideration. Some procedural techniques were used to diminish this potential, such as making participation voluntary and anonymous, using a confidentiality statement, and providing clear instructions to enhance truthful responding and minimize socially desirable responding. The structure of the questionnaire was designed to enhance question clarity and reduce response bias.

## 3. Results

Table 1 presents the demographic and internship-related characteristics of nurse interns. More than half of the participants were aged 22–24 years (52.4%), and females represented the majority of the sample (77.2%). Most participants were single (80.0%) and lived in urban areas (61.4%). Regarding clinical training areas, maternal and newborn health nursing was the most common area (35.9%), followed by medical-surgical nursing (28.3%).

Table 2 summarizes the mean scores and standard deviations of the study variables among nurse interns. The mean professional identity score was 57.84 ± 8.96. The mean dispositional hope score was 47.20 ± 5.68, with mean scores of 23.36 ± 3.44 for agency and 23.84 ± 2.24 for pathways. The mean perceived work environment score was 138.42 ± 18.92. Descriptive statistics for the dimensions of professional identity, dispositional hope, and perceived work environment are presented in Table 2.

Table 3 demonstrates statistically significant positive correlations among the study variables. Perceived work environment was positively correlated with professional identity (r = 0.58, *p* < 0.001) and hope (r = 0.52, *p* < 0.001). Professional identity was also positively correlated with hope (r = 0.49, *p* < 0.001).

Multiple linear regression analysis was performed to examine the adjusted associations between dispositional hope and perceived work environment, professional identity, and selected demographic and internship-related characteristics among nurse interns (Table 4). The overall model was statistically significant, F (10, 1439) = 70.054, *p* < 0.001, with R = 0.572, R^2^ = 0.327, and adjusted R^2^ = 0.323. Perceived work environment (β = 0.359, *p* < 0.001) and professional identity (β = 0.282, *p* < 0.001) showed significant positive associations with dispositional hope after adjustment for the included covariates. In contrast, age category (<22 or >24 years versus 22–24 years), gender, marital status, residence, and the clinical training area contrasts were not significantly associated with dispositional hope (all *p* > 0.05). Overall, perceived work environment and professional identity remained significantly associated with dispositional hope within the adjusted regression model.

## 4. Discussion

The mean professional identity score that the current study registered on the Professional Identity Scale for Nursing Students (PISNS) was 57.84 ± 8.96 (17 items, 1–5 response scale, 17–85 scoring range) and corresponds to a mean of 3.40 for the items. This result corresponds to that of Sana et al. [34] on the professional identity of nursing students. This result also corresponds to the mean of 3.30 ± 0.51 reported by Tu et al. [35] for Chinese nursing interns, and the mean of 3.45 ± 0.77 reported by Hussien et al. [4] for Egyptian nurse interns, employing a similar five-point scale. The proximity of these estimates (3.40, 3.30, 3.45) indicates a relative consistency of professional identity of nurse interns across samples and different professional identity assessment tools. Zeng et al. [36] reported a mean of 4.02 ± 0.63 for Chinese nursing students, using Brown’s Professional Identification Scale, a related but different tool, and due to differences in tools and sample characteristics (undergraduate students vs. interns), the findings are not comparable. Sana et al. [34] reported a total score of 87.07 ± 14.129 on the Professional Identity Five Factors Scale for Pakistani nursing students; this scale, being different in item content, factor structure, and scoring range from the PISNS, also does not allow for a direct comparison. Gadallah et al. [7] and Abd Elhamed and Elborai [37] described findings on professional identity using percentages (86.46% and 78.3%, respectively) without providing group means and standard deviations. Therefore, a direct comparison of professional identity scores is not possible. Among the available studies, the mean score of 3.40 in the present study is in line with the PISNS-based scores from the samples of nurse interns in Egypt [4] and China [35]. Due to the differences in instruments and reporting formats, a comparison with these studies is not appropriate.

The present study found 138.42 ± 18.92 as the mean perceived work environment score. In comparative terms, the absolute value is dependent on the scale, and the same or similar tool is needed to interpret the score. Gadallah et al. [7] evaluated the work environment for a similar nursing population using a self-designed tool and reported the results as percent distribution (81.47%; rated their work environment positively); however, they did not provide mean values of continuous groups, and therefore, their reporting structure does not enable a numeric comparison to the score provided here. The other studies on the work environment [38,39,40,41] used different tools with different scoring ranges; if the tool used to obtain the score was different from the one used in this study, direct comparison of the total score may misrepresent the results in the absence of proportional/standardized metrics, which were not provided in the given studies. The score of 138.42 ± 18.92 will, therefore, be interpreted with the scoring range and norms of the tool used, and in the future, comparisons should be made with studies that used the same validated tool.

The present study has found the mean score for the Adult Hope Scale (AHS) to be 47.20 ± 5.68. The AHS [24] consists of eight items (four from the agency subscale and four from the pathways subscale), each of which is scored from 1 to 8, with a total score range of 8 to 64. In the present study, the mean score per item was 5.90. Hussien et al. [4], in their study on Egyptian nurse interns, reported a hope for the future score of 5.50 ± 1.85 (maximum score related to hope for the future = 8) using a different two-subscale structure (agency: M = 5.50 ± 1.80; pathway: M = 5.50 ± 2.00). Due to the fundamental difference in the scoring ceiling of the two instruments (8 vs. 64), the two scores cannot be compared, although both study samples scored above midpoint in their respective scoring. On the other hand, Yanık and Ediz [42] used the hope item from the Turkish Statistical Institute’s Life Satisfaction Survey to report among 326 Turkish nurses a mean hope score of 2.22 ± 0.95, which is a single-item measure and, thus, lacks a broad range for comparison of hope level, as with the AHS.

Sarioğlu and Kabataş [43] examined mobbing and retained hope in a cross-sectional study of 246 Turkish nurses, reporting a mean total of 21.61 ± 7.98 on their Hope Scale. However, since they did not report the scoring range for the Hope Scale, we do not know where this mean falls on the scale, affecting the comparability with the total AHS in this study. Hope was assessed by Shaheen et al. [44] in 443 undergraduates, of which 9 (2.0%) were interns, using the Herth Hope Index (HHI; Herth, 1992; 12 items, scored 1–4, range 12–48), and they reported a mean percent score of 74.5 ± 10.9. Differences in the HHI and AHS in terms of count, structure, and ceiling scores, and the subsample being predominantly pre-intern, limit the comparability of these studies to the present intern population. The considerable difference in instruments used in studies of dispositional hope in nursing interns indicates that future studies should utilize the AHS to provide a basis for valid numerical comparisons across samples.

The study found positive correlations among perceived work environment, professional identity, and dispositional hope among nurse interns (r = 0.49–0.58). Higher perceived work environment scores were associated with higher professional identity and dispositional hope scores, while higher professional identity scores were also associated with higher dispositional hope scores. These findings are consistent with Gadallah et al. [7], Saker et al. [11], and Basiony et al. [45], who reported positive associations between clinical or work-environment characteristics and professional identity. Differences from findings reported by Hussien et al. [4] and other studies may reflect variation in healthcare systems, supervision, organizational culture, participant characteristics, and methods. Longitudinal and interventional studies are needed to clarify the mechanisms linking work environment, professional identity, and dispositional hope during the transition to professional practice.

Multiple linear regression analysis showed that perceived work environment and professional identity were both significantly associated with dispositional hope after adjustment for demographic and internship-related characteristics. The overall model was statistically significant and explained 32.7% of the variance in dispositional hope (R^2^ = 0.327; adjusted R^2^ = 0.323). Perceived work environment showed a somewhat larger standardized association with dispositional hope (β = 0.359, *p* < 0.001) than professional identity (β = 0.282, *p* < 0.001). However, this difference reflects the relative magnitude of the associations within the specified model and should not be interpreted as evidence of greater causal or substantive importance. Age category, gender, marital status, residence, and clinical training area were not significantly associated with dispositional hope.

The existing literature supports the positive association of professional identity and dispositional hope. Similar to our findings, Hussien et al. [4] found that professional identity and hope were positively correlated for nurse interns. Qiu et al. [46] found that hope as a component of psychological capital was positively related to professional identity, and that hope circumscribed the relationship of workplace violence and professional identity. It appears that professional identity is an important predictor of hope and other future-oriented psychological resources, and the transition to professional nursing practice.

This relation of perceived work environment and dispositional hope is similar to other studies on the impact of the clinical environment and its role in the development and engagement of nurses’ careers. Heldal et al. [47] mentioned that supportive work environments help develop the professional identity of nurses and strengthen their engagement with the nursing profession. Hussien et al. [4] studied the internship nurses’ professional identity and the hope and wellness they developed. Alshareef and Flemban [48] studied the impact of work environment and clinical training on the healthcare trainees’ professional expectations. In this regard, supporting professional and environmental factors is essential to understand the dispositional hope of nurse interns.

The findings also support the study’s theoretical framework that integrates Snyder’s Hope Theory and Social Cognitive Theory. In the context of supportive clinical environments, some resources, feedback, and interpersonal experiences can help develop professional identity and be associated with future-oriented thinking. Also, professional identity can be an instrumental component of the motivational system and goal-directed thinking. However, because of the nature of the study, it is not possible to infer temporal precedence and causation.

These results may help develop Nursing Internship Programs. Suggested additions could include structured mentoring with supportive feedback, opportunities to learn through reflection, observation of professional behavior, and clinical preceptors. The organization may also enhance supportive supervision, purposeful communication, appreciation, and more purposeful clinical learning opportunities. These strategies were not assessed in the present study and therefore are suggestions or implications and not proven interventions. To determine whether these interventions improve professional identity, dispositional hope, and transition-to-practice outcomes, further longitudinal and interventional studies are required.

### Strengths and Limitations

Several limitations should be considered when interpreting the findings of this study. First, the use of self-reported questionnaires may have introduced response bias, including social desirability bias, despite efforts to ensure anonymity and confidentiality. Second, the cross-sectional design limits the ability to determine causal relationships between perceived work environment, professional identity, and dispositional hope; therefore, the findings should be interpreted as associations rather than causal effects. Third, although a total population approach was used to invite all eligible nurse interns, participation was voluntary, and nonresponse may have introduced potential selection bias.

In addition, the study was conducted in university-affiliated hospitals, which may limit the generalizability of the findings to nurse interns working in other healthcare settings, such as private hospitals or non-university institutions. Furthermore, some potentially influential factors were not examined, including previous clinical experience, quality of supervision, preceptor–intern relationships, and individual motivational characteristics, which may contribute to variations in professional identity and dispositional hope. Future research using longitudinal designs, multiple healthcare settings, and additional objective or qualitative data sources is recommended to deepen understanding of the factors influencing nurse interns’ professional development and future expectations.

Additionally, because all variables were measured using self-reported questionnaires administered to the same participants at a single time point, common rater effects and response biases cannot be entirely ruled out. Participants’ current emotional states may have influenced their perceptions and responses. Furthermore, conceptual overlap between certain aspects of the perceived work environment (e.g., organizational belonging and commitment) and professional identity may contribute to shared variance between the constructs. Although statistical procedures were applied to support construct differentiation, residual confounding may remain. Finally, the cross-sectional design prevents determination of causal relationships or the directionality of associations among study variables. Additionally, because clinical unit and supervisor identifiers were not retained, potential clustering effects among nurse interns receiving training within the same units or under similar supervision could not be examined. Future studies should consider multilevel analytical approaches to account for potential dependence among participants and to provide more accurate estimates of contextual effects.

An additional limitation concerns the measurement of perceived work environment. The Perceived Work Environment Questionnaire was originally developed within an employee work-environment context, and some of its dimensions, particularly salary, promotion opportunities, organizational satisfaction, and professional advancement, may have a different or more limited conceptual relevance for nurse interns. Although the questionnaire demonstrated strong internal consistency and a 12-factor structure in the present sample, these psychometric findings do not establish content validity specifically for nurse interns. In addition, the primary analyses used the overall questionnaire score as a composite measure of perceived work environment. While this approach is consistent with the established scoring procedure and the overall study aim, the multidimensional structure of the instrument indicates that the 12 domains represent distinct components of the work environment. Consequently, aggregation across these domains may obscure potentially important domain-specific associations with professional identity and dispositional hope. Future studies should undertake formal content-validity evaluation of the instrument in nurse intern populations and examine the individual dimensions separately, as well as evaluate whether a higher-order measurement model is psychometrically supported.

## 5. Conclusions

This study demonstrated significant positive associations among perceived work environment, professional identity, and dispositional hope among nurse interns. A more favorable perceived work environment was associated with stronger professional identity and higher dispositional hope, while professional identity was also positively associated with dispositional hope. In the adjusted regression model, both perceived work environment and professional identity remained independently associated with dispositional hope, whereas age category, gender, marital status, and critical care exposure were not significantly associated with hope. These findings highlight the relevance of both environmental and professional factors during the transition from nursing education to professional practice.

The findings support the importance of strengthening internship environments through effective supervision, constructive feedback, professional recognition, mentoring, and meaningful clinical learning opportunities. Strategies that promote professional identity development, including role modeling and reflective learning, may also support nurse interns’ confidence and future-oriented expectations. However, because of the cross-sectional design, the observed relationships should be interpreted as associations rather than causal effects. Future longitudinal and intervention studies are recommended to examine the direction and mechanisms of these relationships and to determine whether improvements in the work environment and professional identity can enhance dispositional hope during the transition to professional nursing practice.

## Figures and Tables

**Table 1:** Distribution of Nurse Interns According to Their Personal and Job Characteristics (*n* = 1450).

Characteristics	Categories	N	%
Age (years)	<22	420	28.9
	22–24	760	52.4
	>24	270	18.6
Gender	Male	330	22.8
	Female	1120	77.2
Marital status	Single	1160	80.0
	Married	290	20.0
Residence	Urban	890	61.4
	Rural	560	38.6
Current Clinical Training Area	Medical-Surgical Nursing	410	28.3
	Maternal and newborn health nursing	520	35.9
	Critical Care	260	18.0
	Dialysis and Emergency	260	17.9

**Table 2:** Mean Scores of Study Variables among Nurse Interns (*n* = 1450).

Study Variables	Mean ± SD
Professional Identity	57.84 ± 8.96
Professional self-image	21.12 ± 3.36
Retention & turnover risk	13.48 ± 2.41
Social comparison	10.26 ± 2.08
Career independence	7.02 ± 1.41
Social modeling	5.96 ± 1.52
Dispositional Hope	47.20 ± 5.68
Agency	23.36 ± 3.44
Pathways	23.84 ± 2.24
Perceived Work Environment	138.42 ± 18.92
Job satisfaction	10.92 ± 1.99
Recognition	10.08 ± 2.11
Work itself	14.36 ± 3.28
Opportunities for promotion	9.24 ± 2.37
Professional advancement opportunities	16.84 ± 4.11
Responsibility	14.92 ± 2.64
Good feelings about organization	9.18 ± 2.83
Clarity of mission	8.46 ± 2.09
Relationship with co-workers	13.72 ± 3.06
Effective supervisor	17.64 ± 4.08
Salary	6.12 ± 1.36
Presence of core values	6.94 ± 1.42

Note: Subscale scores are presented descriptively and should not be directly compared across domains because the subscales contain different numbers of items.

**Table 3:** Correlation Between Perceived Work Environment, Professional Identity, and Dispositional Hope Among Nurse Interns (*n* = 1450).

Variables	1	2	3
1. Perceived Work Environment	1		
2. Professional Identity	0.58 **	1	
3. Dispositional Hope	0.52 **	0.49 **	1

Note: r = Pearson’s correlation coefficient, ** *p* < 0.001.

**Table 4:** Multiple Linear Regression Analysis of Factors Associated with Dispositional Hope among Nurse Interns (*n* = 1450).

Independent Variables	B	SE	β	95% CI	t	*p*
Perceived Work Environment	0.108	0.008	0.359	0.092 to 0.124	13.498	<0.001
Professional Identity	0.179	0.017	0.282	0.146 to 0.212	10.625	<0.001
Age < 22 vs. 22–24	−0.848	1.495	−0.068	−3.781 to 2.084	−0.568	0.570
Age > 24 vs. 22–24	−0.030	1.811	−0.002	−3.582 to 3.521	−0.017	0.987
Male vs. Female	0.142	0.583	0.010	−1.001 to 1.285	0.243	0.808
Married vs. Single	−0.249	1.090	−0.018	−2.387 to 1.889	−0.228	0.819
Rural vs. Urban	0.309	0.771	0.026	−1.203 to 1.820	0.401	0.689
Medical-Surgical vs. Maternal/Newborn	1.174	1.569	0.093	−1.904 to 4.252	0.748	0.454
Critical Care vs. Maternal/Newborn	−0.613	0.802	−0.041	−2.186 to 0.960	−0.765	0.445
Dialysis/Emergency vs. Maternal/Newborn	0.544	1.706	0.037	−2.803 to 3.892	0.319	0.750
R = 0.572 | R^2^ = 0.327 | Adj R^2^ = 0.323 | F (10, 1439) = 70.054 | *p* < 0.001						

## Data Availability

The datasets used and/or analyzed during the current study are available from the corresponding author on reasonable request.

## Abbreviations

ISPIN	The International Society for Professional Identity in Nursing
PISNS	Professional Identity Scale for Nursing Students
AHS	Adult Hope Scale
SPSS	Statistical Package for the Social Sciences
VIF	Variance Inflation Factor
